# Blood-Letting in Mental Disorders

**Published:** 1854-07

**Authors:** Pliny Earle


Blood-Letting in Mental Disorders.—By Pliny Earle, M. D. —Dr. Earle, in reply to the question, "To what extent, in regard to both frequency and quantity, is the abstraction of blood required in the treatment of Insanity," makes the following remarks, (in *Amer. Jour. Insanity*, (for May, 1854,):—

"A reply to the proposition at the commencement may now be attempted. It is evident, however, from the very nature of the case, that no positive, definite answer, couched in terms as fixed as figures represent numbers, can be given. It must be merely approximative. I shall endeavor to convey it in a series of facts, truths or inferences, which I hope are fairly deduced from the substance of the foregoing pages.

1. Insanity, in any form, is not, of itself, an indication for blood-letting.

2. On the contrary, its existence is, of itself, a contra-indication. Hence, the person who is insane should, other things being equal, be bled less than one who is not insane.

3. The *usual* condition of the brain, in mania, is not that of active inflammation, but of a species of excitement, irritability, or irritation, perhaps more frequently resulting from or accompanied by anæmia, debility, or abnormal preponderance of the nervous over the circulatory functions, than in connection with plethora and enduring vital power.

4. The excitement, both mental and physical, produced by this irritation, can, in most cases, be permanently subdued, and its radical source removed by other means, more readily than by bleeding.

5. Yet insanity may be co-existent with conditions,—such as positive plethora, a tendency to apoplexy or paralysis, and sometimes sthenic congestion or inflammation, which call for the abstraction of blood.

6. Venesection in mental disorders should not be absolutely abandoned, although the cases requiring it are very rare.

7. As a general rule, *topical* is preferable to *general* bleeding.

8. In many cases where the indication for direct depletion is not urgent, but where blood-letting, particularly if local, might be practiced without injury, it is safer and better to treat by other means, equalizing the circulation and promoting the secretions and excretions.

9. The physical conditions requiring blood-letting more frequently exist in mania than in any other of the ordinary forms of mental alienation.

10. Insanity following parturition, other things being equal, is to be treated by bleeding less frequently than that which has its origin in other causes.

11. If the mental disorder be the direct result of injury to the head, the treatment must be directed to the wound, or its physical effects, not specially to the psychic condition.

12. In many cases where insanity is accompanied by typhus symptoms, and in some where the aspect is that of acute phrenitis, active stimulants alone can save the patient, and direct depletion from the circulation is almost certainly fatal."

